# Investigation of Vitamin D Levels in Men with Suspected Infertility

**DOI:** 10.3390/life14020273

**Published:** 2024-02-18

**Authors:** Fırat Aşır, Senem Çetin Duran, Muhammet Afşin, Enis Duran, Tuğcan Korak, Fırat Şahin

**Affiliations:** 1 Department of Histology and Embryology, Medical Faculty, Dicle University, 21280 Diyarbakır, Turkey; senem.duran@dicle.edu.tr (S.Ç.D.); sfirat9021@gmail.com (F.Ş.); 2 Department of Andrology, Health Sciences University, Gazi Yasargil Education and Research Hospital, 21090 Diyarbakir, Turkey; muhametafsin@gmail.com; 3 Department of Gynecology and Obstetrics, Medical Faculty, Van Yüzüncü Yıl University, 65000 Van, Turkey; enis.duran@dicle.edu.tr; 4 Department of Medical Biology, Medical Faculty, Kocaeli University, 41001 Kocaeli, Turkey; tugcankorak@gmail.com

**Keywords:** vitamin D, sperm motility, infertility, functional enrichment

## Abstract

Male infertility may be caused by an impaired sperm functionality, with insufficient vitamin D levels affecting the quantity and development of motile sperm. Given the influence of vitamin D on vital aspects of male infertility, this study aimed to investigate the correlation between vitamin D levels and male infertility, along with exploring the possible mechanism of action. A total of 306 male participants were included. Semen samples were collected and analyzed for semen parameters with demographic features. Patients were classified into two groups based on vitamin D levels of <20 ng/mL (low) and ≥20 ng/mL (high). The Super-PRED, Swiss TargetPrediction, GeneCards, and DisGeNET databases were utilized to retrieve potential molecular targets associated with both vitamin D and male infertility, while the STRING database was employed for constructing protein–protein interaction (PPI) networks and conducting a functional enrichment analysis. A total of 146 patients (47.71%) showed low vitamin D levels and 160 patients (52.29%) had high vitamin D levels. Vitamin D was not strongly influenced by demographic parameters. Vitamin D demonstrated significant positive correlations with type A and B sperm motility. Conversely, it exhibited significant negative correlations with type C and D sperm motility. Hormones (thyroid-stimulating hormone, follicle-stimulating hormone, prolactin, luteinizing hormone, estradiol) were not significantly associated with vitamin D; however, testosterone was significantly positive correlated with vitamin D. Notably, no significant correlation was found between vitamin D levels and iron, ferritin, hemoglobin, hematocrit, calcium, magnesium, and phosphorus levels. The functional annotations of potential vitamin D targets associated with male infertility primarily indicated involvement in regulating infection, the immune response, forkhead box O (FOXO) and hypoxia-inducible factor 1 (HIF1) signals in male infertility. Adequate vitamin D levels are associated with an improved reproductive health, evidenced by positive correlations with hormone levels and sperm motility. Specifically, the FOXO and HIF-1 signaling pathways may be effective in the potential molecular mechanisms underlying the impact of vitamin D on male infertility and/or in the significant correlations identified.

## 1. Introduction

Infertility is a common problem affecting approximately 70 million people around the world. An estimated 9% of couples face fertility problems, with 50% of these issues attributed to male factors [1]. Although lifestyle, nutrition, and genetics are common risk factors for male infertility, and most cases are idiopathic, the underlying causes are still not identified or understood [2]. Moreover, sperm abnormalities are also one of the causes of male infertility, comprising roughly 30% of cases [3]. Hormonal changes induced by nutritional factors are particularly associated with male infertility [4,5]. In recent years, vitamin D has been a prominent subject of study due to its pleiotropic role, encompassing autocrine, paracrine, and endocrine functions on various target organs and systems [6,7]. Belonging to the group of secosteroids, its primary activity involves the regulation of calcium and phosphorus homeostasis. Biologically, the two most significant members of the vitamin D group are ergocalciferol (vitamin D2) and cholecalciferol (vitamin D3) [8]. Vitamin D exerts its various biological effects through binding to vitamin D receptors (VDRs) [9]. Vitamin D-metabolizing enzymes (such as cytochrome P450 mixed-function oxidases [10]) and VDRs are concurrently present in various cells within the male reproductive system, including Sertoli cells, germ cells, Leydig cells, spermatozoa, and the epithelial cells of the male reproductive tract. This suggest that local vitamin D responses can be regulated within the reproductive male system. Specifically, the cells in the testes appear capable of synthesizing and breaking down vitamin D locally, independently of systemic vitamin D metabolism. Moreover, the expression of VDR in the testis suggests a potential autocrine and paracrine role for vitamin D, influencing testicular function and potentially impacting male infertility [11]. While the association of vitamin D with the cytochrome P450 family 24 subfamily A (CYP24A) enzyme and cAMP/PKA pathways has been identified as playing an important role in spermatogenesis and sperm motility, there is still a lack of sufficient data concerning the mechanism of action of vitamin D in the context of the male genital system. Consequently, numerous aspects remain unclear to date [12]. 

Vitamin D deficiency, delineated by serum levels below 20 ng per milliliter (<50 nmol/L), exhibits a pervasive global prevalence, yet its health effects remain unclear [6]. The role of vitamin D in the male reproductive process has been underscored, considering the identified expression of enzymes responsible for metabolizing vitamin D in the testis and spermatozoa [13]. Sood et al. [14] investigated the impact of vitamin D deficiency on spermatogenesis. They found reduced levels of testicular glutamyl transpeptidase activity in rats with vitamin D deficiency, indicating it as an indicator of Sertoli cell function. Akhavizadegan et al. [15] studied 230 male participants and observed that vitamin D levels were significantly lower in infertile men. Numerous studies have demonstrated the pivotal role of vitamin D in maintaining male reproductive system health, showing its capacity to influence crucial factors including spermatogenesis, sperm motility, and hormonal regulation [16].

Considering the global prevalence of infertility with the significant contribution of male factors, and the impact of vitamin D on male spermatogenesis, sperm motility, and hormonal regulation, this study aimed to investigate the potential correlation between vitamin D levels and male infertility, and also explore the potential mechanism of action underlying this association.

## 2. Materials and Methods

### 2.1. Research Design

This study was designed as a prospective cohort study. The ethical committee approval for the study was obtained from the Clinical Research Ethics Committee, Gazi Yaşargil Training and Research Hospital, Health Sciences University (date: 14 January 2022, approval number: 2022/4). All participants were informed about the study and agreed and signed patient consent forms. The study was performed in accordance with the Declaration of Helsinki.

### 2.2. Patient Selection and Sampling

A total of 306 male individuals with idiopathic infertility were enrolled in this study after being admitted to the infertility clinic at Gazi Yaşargil Training and Research Hospital between September 2022 and March 2023. Men were between the ages of 20 and 48, with at least one year of infertility complaints. All couples were healthy and had no previous (acute/chronic) disease or surgery history in the past or during admission. Women in the couples did not have any female problems (menstrual irregularity, hormonal disorder, chronic disease, tubal obstruction). Participants who previously had a history of infertility or supplement treatment were also excluded. The sample size was determined by using Gpower by Cohen’s criteria. A sample size analysis was conducted with an effect size of 0.15 with 80% power and an α error of 0.05.

### 2.3. Demographic Parameters 

Socio-demographic characteristics (age, education level, urban/rural life, occupation, number of children, consanguineous marriage) of all participants were extracted from survey. Body mass index (BMI) was determined by measurements of the height and weight of each person by medical personnel. Abstinence time was self-recorded by participants. 

### 2.4. Semen Sample Collection and Analysis

Semen samples were collected after 2–7 days of sexual abstinence from participants for semen analysis. Samples were incubated for 30–60 min in an incubator at 37 °C and evaluated macroscopically and microscopically under a light microscope (Olympus CX31). Semen parameters such as sperm concentration (million/mL), semen volume (mL), total sperm count (million/mL) and total count of progressive motile sperm (million/mL) were recorded. Sperm concentration was measured as sperm count in 1 mL of ejaculation. Total sperm count was measured by number of sperm in total ejaculate. Sperm motility of patients was divided into three categories: progressive (type A and B), non-progressive (type C) and immotile (type D). Sperm count was measured by summation of type A and type B + type C + type D in 10 cells under a light microscope. Total number of progressive motile sperm was calculated by number of progressive motile (%) × total sperm count.

### 2.5. Measurement of Laboratory Parameters

Blood samples were collected from the participants on the same day as semen samples in the morning hours. Hormone levels and biochemical-hematological parameters were recorded for each participant. Blood samples were centrifuged at 4000× *g* rpm for 15 min, the blood serum was analyzed for FSH, LH, total testosterone, TSH, prolactin, E2, folic acid, vitamin D, ferritin, calcium (Ca), magnesium (Mg), phosphorus (P), iron (Fe), hemoglobin and hematocrit by Cobas 6000 (Roche Diagnostic, Mannheim, Germany). All measurements were done with immunoassay commercial kits (Elecsys^®^, Roche, Germany coefficient of correlation ≥ 0.95).

### 2.6. Identification of Male-Infertility-Associated Molecular Targets of Vitamin D and Functional Enrichment Analysis

The mechanisms that could explain the significant results associated with vitamin D were analyzed through database scanning and bioinformatic approaches. The Super-PRED (https://prediction.charite.de/index.php, accessed on: 10 November 2023), Swiss TargetPrediction (http://www.swisstargetprediction.ch, accessed on: 10 November 2023), and GeneCards (https://www.genecards.org/, accessed on: 10 November 2023) databases were queried for 25-hydroxyvitamin D3 and/or its SMILES (Simplified Molecular Input Line Entry System), chemical structure line notation (a typographical method using printable characters) for chemical formula which was retrieved from PubChem (https://pubchem.ncbi.nlm.nih.gov/, accessed on: 10 November 2023) to identify vitamin D target proteins. On the other hand, target proteins associated with male infertility were obtained from DisGeNET (https://www.disgenet.org/, accessed on: 15 November 2023) and GeneCards databases. Next, a Venn diagram was constructed based on the Uniprot IDs using the jvenn tool to identify intersecting targets between vitamin D and male infertility [17]. The protein–protein interaction (PPI) network of common target proteins was established through the Search Tool for the Retrieval of Interacting Genes/Proteins (STRING; https://string-db.org, accessed on: 15 November 2023) with a confidence level of 0.400. In the STRING analysis, false discovery rate (FDR) values were sorted from low to high. An FDR value below 0.05 was regarded as statistically significant. The functional enrichment analysis utilized the top 10 data retrieved from STRING for Gene Ontology (GO) biological processes (BPs), molecular functions (MFs), cellular components (CCs), and Kyoto Encyclopedia of Genes and Genomes (KEGG) pathways, which were visualized using SRplot [18].

### 2.7. Statistical Analysis

All statistical analyses were performed using IBM SPSS for Windows version 20.0 (IBM Corp., Armonk, NY, USA). A Kolmogorov–Smirnov test was used to assess the normality distribution. Since the data were distributed non-normally, continuous variables were presented as medians and interquartile range (IQR). Categorical variables were summarized as frequencies and percentages. Comparisons between groups were carried out using the Mann–Whitney U test. Correlation between categorical variables were tested by the Chi-square test. The relationship between continuous variables was analyzed via Spearman’s correlation analyses. A *p*-value of <0.05 was considered statistically significant.

## 3. Results

### 3.1. General Characteristics of Participants

The general characteristics of the whole study sample are listed in Table 1. 

Relations of demographic parameters with vitamin D are shown in Table 2. There were no significant relations between vitamin D and education, job, location, infertility type, consanguineous marriage and number of children (*p* > 0.05). This shows that demographic parameters may not be strong determinants of vitamin D status in the population of this study.

We categorized the participants into two groups based on their vitamin D levels. Patients with a vitamin D level < 20.00 ng/mL were categorized as low level, while a vitamin D level ≥ 20 ng/mL was the high level. The following analyses were performed between these two groups. Age, marriage and childless years and BMI of participants are shown in Table 3 by vitamin D levels (low or high). Out of 306 patients, 146 (47.71%) had low vitamin D levels, while 160 (52.29%) had high levels. There was no significant change between groups in terms of age, marriage, childless years and BMI parameters (*p* > 0.05), suggesting the vitamin D level is possibly independent of these parameters.

### 3.2. Semen Analysis of Patients

Table 4 shows the parameters of semen analysis. The median values of sperm concentration and total count of progressive motile sperm were non-significantly higher in participants with high vitamin D, while median of semen volume and total sperm count were non-significantly higher in participants with low vitamin D. The median percentage of type A and B motility in high vitamin D group was significantly higher than in low vitamin D group. This may show that higher vitamin D level with better sperm motility. Median percentage of Type C motility were non-significantly higher in low vitamin D group than in high vitamin D group. Type D motility was significantly higher in patients with low vitamin D compared to patients with high vitamin D group, suggesting low vitamin D may indicate poorer sperm quality. Abstinence time showed non-significant change between patients with low and high vitamin D.

### 3.3. Hormonal Analysis of Patients

Hormones and folic acid values of participants are shown in Table 5. The median of TSH and FSH in the low vitamin D group was non-significantly higher than that in high vitamin D group. However, the median of LH, E2, testosterone and folic acid in the high vitamin D group was non-significantly higher than that in low vitamin D group. Only the difference in the median of prolactin showed significance between groups, and it was higher in the high vitamin D group compared to the low vitamin D group. These results suggest that vitamin D levels may not strongly influence these hormone levels in the studied population, except for prolactin.

### 3.4. Blood and Essential Minerals of Patients 

Iron, calcium, magnesium and phosphorus levels were high in patients with a high level of vitamin D. Low vitamin D was observed in patients with only a high ferritin level. The medians of hemoglobin and hematocrit were same for groups. The difference in the median of these parameters did not show significance between high and low vitamin D levels (Table 6).

### 3.5. Correlation Analyses of Demographic and BMI Parameters

Table 7 shows that vitamin D levels did not appear to be significantly influenced by age, marital status and BMI or in the studied population (*p* > 0.05).

### 3.6. Correlation Analyses of Semen Parameters

The correlation analysis of vitamin D levels (low or high) and the semen parameters are shown in Table 8. Vitamin D was not correlated with total sperm concentration, semen volume, total count of progressive motile sperm or abstinence time (*p* > 0.05). Vitamin D was significantly positively correlated with type A and B motility while it was significantly negatively correlated with type C and D motility. This may suggest that high vitamin D levels might be associated with high motility of sperm, which is important for fertility. A higher percentage of type D motility in patients with low vitamin D levels might indicate poorer sperm quality or lower fertility potential. Sample variability and complexity of semen parameters might cause low Spearman rank correlation coefficients, although motility parameters showed significance between groups. 

### 3.7. Correlation Analyses of Hormonal Parameters

The correlation analysis between vitamin D and hormones and folic acid is shown in Table 9. Vitamin D was significantly positive correlated with testosterone. This shows that individuals with higher levels of vitamin D may also have higher levels of testosterone. This association suggests that vitamin D might play a role in influencing testosterone production or regulation. The low Spearmen correlation coefficient shows that the mechanism of action of vitamin D and testosterone may not directly overlap. Vitamin D had no significant correlation with TSH, FSH, LH, Prolactin, E2 or folic acid. 

### 3.8. Correlation Analyses of Iron and Essential Mineral Parameters

Table 10 shows the correlation between vitamin D and iron-related parameters and essential minerals. There is no significant correlation between vitamin D levels and iron, ferritin, hemoglobin, hematocrit, calcium, magnesium, or phosphorus (*p* > 0.05) (Table 10). The lack of significant correlation with iron-related parameters (iron, ferritin, hemoglobin, hematocrit) indicates that vitamin D levels may not be strongly associated with iron metabolism or overall red blood cell health. The absence of significant correlations with essential minerals suggests that variations in vitamin D levels may not have a statistically significant impact on the levels of these minerals. 

### 3.9. Male-Infertility-Correlated Molecular Targets of Vitamin D and Their Functional Annotations

**Figure 1:**
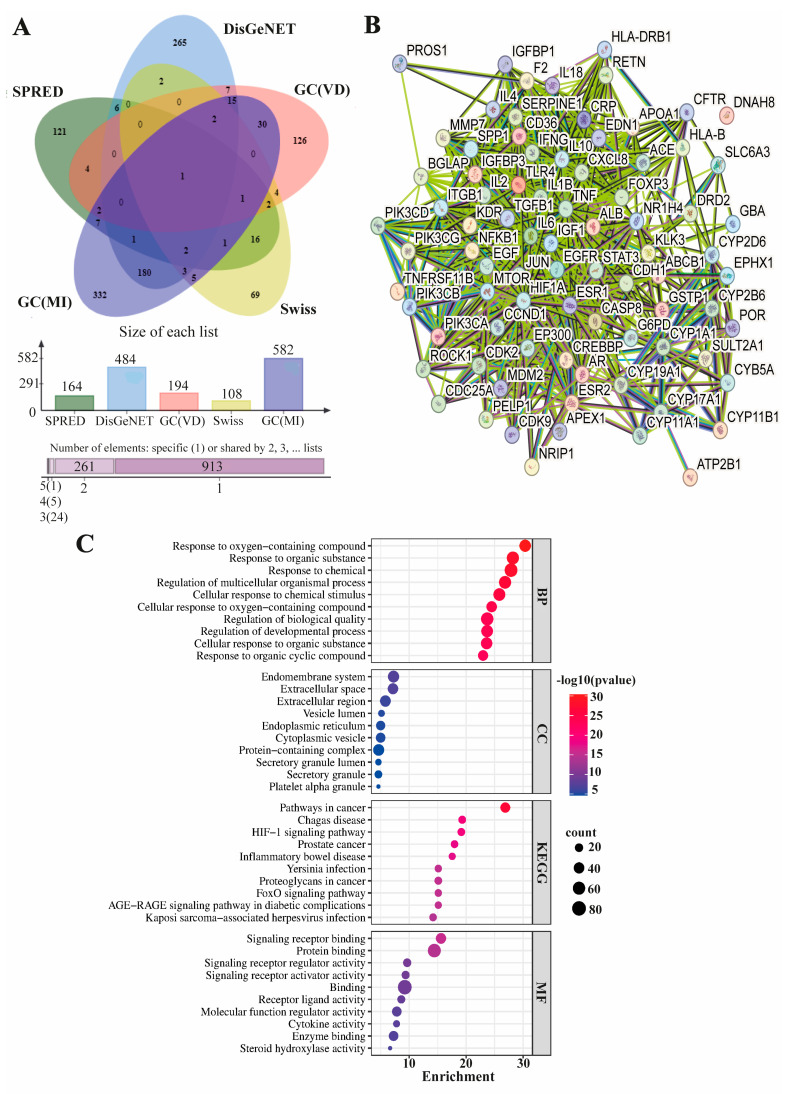
Molecular targets of vitamin D associated with male infertility and their functional annotations. (**A**). The Venn diagram illustrates the proteins that are common and unique among databases covering both vitamin D and male infertility targets. The graph below represents the count of proteins observed in the databases. (SPRED: Super-PRED, GC: GeneCards, VD:Vitamin D, MI: Male infertility.) (**B**). A PPI network was constructed using the 85 proteins that overlap between vitamin D targets and those intersecting with male infertility. The FDR of <0.05 was considered statistically significant. (**C**). KEGG analysis and BP, CC and MF in GO analysis revealed the relationship between top 10 potential male-infertility-associated vitamin D targets and functional pathways. Red dots denote the most significant processes, whereas blue indicates lower significance, as determined by −log10 (*p*-value). Larger dots on the graph represent a higher number of involved genes.

The results derived from the Super-PRED, Swiss TargetPrediction, and GeneCards databases indicated the presence of 164, 194, and 108 target proteins for vitamin D, respectively. In the DisGeNET and GeneCards databases, 484 and 582 male-infertility-associated proteins were identified. Consequently, the 85 shared target proteins were obtained from databases encompassing both vitamin D and male infertility targets (Figure 1A). Using the intersecting target proteins, a STRING PPI analysis yielded 82 nodes and 1107 edges (PPI enrichment *p*-value < 1.0 × 10^−16^) (Figure 1B). The functional annotations were then generated by utilizing the top 10 ranked Gene Ontology (GO) and KEGG pathway data from the intersected STRING PPI analysis. In terms of biological processes (BPs), the enrichment primarily centered around responses to oxygen-containing compounds, organic substances, and chemicals, as well as the regulation of multicellular organismal processes and biological quality. Regarding cellular components (CCs), the common targets of vitamin D and male infertility were mainly associated with the endomembrane system, extracellular space/region, vesicle lumen, and endoplasmic reticulum. In molecular functions (MFs), the enrichment of intersected targets predominantly involved signaling receptor binding, protein binding, signaling regulator/activator activity, and receptor ligand activity. The KEGG pathway analysis revealed PPI enrichment in pathways such as cancer, Chagas disease, hypoxia-inducible factor 1 (HIF-1) signaling pathway, inflammatory bowel disease, and forkhead box, subgroup O (FOXO) signaling pathway (Figure 1C).

## 4. Discussion 

This study aimed to investigate correlation between vitamin D levels and male infertility by analyzing demographic parameters, semen parameters, hormones and essential minerals of 306 male participants with infertility suspicion. A bioinformatic analysis was also performed to support potential mechanism of vitamin D and male infertility. Demographic features and vitamin D were not related in our population. A high percentage of type A and B sperm motility was found in patients with higher vitamin D levels, indicating a positive correlation of vitamin D and sperm motility. Low vitamin D levels were also associated with a high percentage of type D motility, suggesting low vitamin D levels may have a role in sperm infertility. Correlation of vitamin D and testosterone was significantly positive, which may show vitamin D level is dependent of testosterone. Vitamin D was not associated with iron related metabolism and essential minerals for our study population. Additionally, the analysis of target proteins indicated potential links between vitamin D and male infertility through various biological processes and pathways, including cancer, HIF-1 signaling, and FOXO signaling.

The etiology of male infertility includes low sperm production, sperm abnormalities, idiopathic factors, genetic background, medications, infections. The exact molecular mechanisms of vitamin D in male reproduction are not fully understood. Although vitamin D receptors and metabolizing enzymes are expressed in the testis and sperm, the role of vitamin D in male fertility is still open to debate [1,11]. Kwiecinsk et al. [19] found that successful mating rates (the presence of sperm in the female’s vaginal canal) of vitamin D-deficient men were reduced by 45% compared to vitamin D-sufficient men. Jensen et al. [20] analyzed serum the vitamin D levels and sperm quality of 300 male patients and discovered a positive correlation between vitamin D levels and sperm quality. The authors stated that vitamin D may promote optimal sperm function. In a review study, the authors found that vitamin D has positive effects on sperm quality and motility. Vitamin D is thought to modulate sperm function parameters in male patients. Maghsoumi-Norouzabad et al. [21] conducted a randomized, triple-masking, placebo-controlled clinical trial on 86 asthenozoospermia infertile men with serum 25 hydroxy vitamin D3 < 30 ng/mL. They found that 3 months of vitamin D supplementation was not associated with demographic features and hormones (FSH, LH, E2), semen volume and count. However, vitamin D significantly increased sperm motility. Similar to other research findings, we found a positive correlation between higher vitamin D levels and improved sperm motility, particularly in terms of progressive motility. Moreover, a low vitamin D level was linked to a high type D sperm motility. Since sperm cells contain VDR, it is possible that vitamin D may have a potential role in sperm functionality. 

Rafiq et al. [22] investigated the association of vitamin D and testosterone hormone in 643 Dutch men. The authors found a positive correlation between D vitamin levels and total and bioavailable testosterone levels, showing a low vitamin D level was observed in men with lower testosterone hormone. Wentz et al. [23] recorded vitamin D levels in 796 males and found those with the lowest vitamin D levels exhibited significantly lower testosterone concentrations compared to those with the highest vitamin D levels. Another study examined the impact of vitamin D supplementation on 62 infertile men with impaired spermatogonia tests. The findings indicated no statistically significant distinctions between the cohort receiving vitamin D3 supplementation and the placebo group concerning spermogram parameters or serum levels of LH, FSH, total testosterone, free testosterone, and free androgen index [24]. The association between testosterone and vitamin D is not consistent in studies due to the age of participants. Testosterone levels decline in men with age, beginning at 30 years [25]. However, in our study, age did not act as an effect modifier. Moreover, the exact mechanism of the hormonal regulation of testosterone production is still unclear [26]; thus, the effect of vitamin D on testosterone needs further investigating.

Hormones, minerals and trace elements are also important for male fertility [27]. Gokalp et al. found that COVID-19 infection was significantly associated with hormones and reduced testosterone levels [28]. Allouche-Fitoussi et al. [29] investigated the role of zinc in sperm function and found that an adequate amount of zinc is required for regular sperm function and proper fertilization. In a study, 30 infertile asthenozoospermic males were included and their semen samples were subjected to trace element analysis. The study revealed that magnesium showed a significant correlation with sperm motility [30]. Many studies showed that calcium deficiency leads to a low sperm motility in the fertilization process [31,32,33]. Although the relation between sperm motility and trace elements is known, little is known of the effect of vitamin D on trace element regarding trace elements and hormones. In our study, a significant correlation was found between vitamin D and testosterone hormones. However, vitamin D was not significantly linked with trace elements (calcium, magnesium, phosphorus), hormones (TSH, FSH, LH, prolactin and E2) or folic acid. Additionally, there was no significance between vitamin D levels and iron-related parameters (iron, ferritin, hemoglobin, hematocrit). This is the first study to show the relation of vitamin D with these parameters, so further studies are required for extensive comprehension.

Beyond biochemical parameters, genes associated with male infertility, involved in processes such as spermatogenesis and the development of the male reproductive system, may lead to qualitative or quantitative sperm defects, accounting for a significant number of infertility cases. However, the genetic etiology in 40% of male infertility cases remains unknown [34]. Although numerous genes have been implicated in male fertility, limited information exists regarding the mechanism of action of vitamin D on the male genital system [12,34]. We attempted to identify male infertility candidate genes potentially associated with vitamin D using bioinformatic approaches. Thus, the potential underlying molecular basis for the obtained significant association between vitamin D levels and clinical parameters in male infertility was evaluated in the current study. 

The functional annotations of potential vitamin D targets associated with male infertility primarily revealed involvement in the response to oxygen-containing and organic compounds, as well as in signaling receptor binding. These findings are in line with in vivo and in vitro studies in the literature indicating the role of vitamin D in signaling and enzyme regulation in male infertility [11,12]. The vitamin D/VDR signaling pathway has been demonstrated to play roles in autoimmunity, cell differentiation, apoptosis, HIF-1 signaling and prevention of oxidative stress, with the involvement of FOXO3 induction [11,35]. HIF-1 has been shown to play essential roles in the process of spermatogenesis and in predicting the extent of apoptosis in spermatozoa [36,37]. Moreover, FOXO was shown to be involved in the development of spermatogonia [38], the maintenance of spermatogonial stem cells [39] and anti-stress activity in the testes [38]. On the other hand, it has been revealed that microbial infection and immune response have an effect on male infertility, in addition to the impacts of impaired spermatogenesis and testicular functions [11,40]. Microbial infection-induced male infertility is primarily accomplished through an immune response, where microorganisms stimulate the accumulation of immune cells, proinflammatory cytokines, and chemokines. Additionally, the production of anti-sperm antibodies and biofilms can also result in damage to germ cells and disruption of the normal spermatogenic function [40]. Our KEGG results integrate the effects of vitamin D with male infertility, indicating the potential effectiveness of regulating infection, immune responses, FOXO, and HIF1 signaling pathways in male infertility. Moreover, it has been observed that effective vitamin D targets in male infertility exhibit similarities with cancer signaling pathways and infection-related diseases.

## 5. Conclusions

The present study highlights a positive association between sufficient vitamin D levels and improved reproductive health, as evidenced by positive correlations with hormone levels and sperm motility. Moreover, our functional enrichment analysis emphasizes potential regulatory mechanisms, particularly involved in the regulation of infection, the immune system, FOXO, and HIF1 signaling pathways, which may play crucial roles in the significant associations identified between vitamin D and male infertility. Future in vitro and in vivo studies could experimentally validate the identified regulatory mechanisms, providing a comprehensive understanding of how these pathways contribute to associations between vitamin D and male infertility. This research may lead to targeted interventions and therapeutic strategies to optimize vitamin D levels for improving male reproductive health.

## Figures and Tables

**Table 1:** Demographic, semen, biochemical and blood parameters of all participants.

Parameters (*n* = 306)	Mean ± SD	Median (Q1–Q3)
Age (years)	31.45 ± 6.96	30.6 (27–36)
BMI (kg/m^2^)	24.95 ± 3.05	24.78 (23.4–27.23)
Marriage (years)	4.58 ± 3.86	4 (2–6)
Years without children (*n*)	3.17 ± 2.67	2 (1–4)
Number of children having (*n*)	0.42 ± 0.74	2 (0–1)
Vitamin D (ng/mL)	20.76 ± 8.09	20.5 (13.6–25.93)
Sperm concentration (million/mL)	38.04 ± 38.32	29 (5.87–60)
Semen volume (mL)	3.01 ± 1.46	2.7 (2–3.77)
Total sperm count (million/mL)	105.08 ± 116.09	70 (13.9–156.25)
Total count progressive motile sperm (million/mL)	57.47 ± 78.20	30 (3–84)
Type A and B motility (%)	46.14 ± 23.89	50 (29.75–65)
Type C motility (%)	8.49 ± 7.15	5 (5–11)
Type D motility (%)	45.58 ± 24.34	41 (28.75–56)
Abstinence time (day)	3.41 ± 0.53	3 (3–4)
TSH (mU/L)	1.74 ± 0.93	1.59 (1.11–2.13)
FSH (U/L)	7.86 ± 7.94	5.74 (3.8–8.53)
LH (U/L)	6.88 ± 4.46	6.14 (4.61–7.85)
Prolactin (µg/L)	16.17 ± 7.35	15.14 (11.23–18.66)
E2 (ng/L)	24.45 ± 10.88	23.06 (17.96–29.82)
Testosterone (ng/mL)	4.74 ± 2.09	4.34 (3.28–5.63)
Folic acid (ng/mL)	7.34 ± 3.12	6.81 (5.23–8.62)
Iron (µg/dL)	97.91 ± 34.91	87.5 (76–113)
Ferritin (µg/L)	120.76 ± 70.09	112 (74–152)
Hemoglobin (g/dL)	16.16 ± 0.84	16.3 (15.67–16.7)
Hematocrit (g/dL)	47.37 ± 2.73	47.6 (45.9–48.9)
Calcium (mg/dL)	9.57 ± 0.49	9.6 (9.37–9.8)
Magnesium (mg/dL)	2.01 ± 0.37	1.97 (1.79–2.17)
Phosphorus (mg/dL)	3.49 ± 0.43	3.42 (3.24–3.69)

SD: standard deviation.

**Table 2:** Demographic of patients and correlation analysis with vitamin D.

Parameters		N (%)	Significance with Vitamin D
Education	Primary education	167 (54.57%)	0.245
	Secondary education	53 (17.32%)	
	Higher education	86 (28.10%)	
Job	Jobless	33 (10.78%)	0.969
	Worker	211 (68.95%)	
	Civil servant	62 (20.26%)	
Location	Rural	72 (23.52%)	0.177
	Urban	234 (76.47%)	
Infertility type	Primary	219 (71.56%)	0.529
	Secondary	87 (28.43%)	
Consanguineous marriage	No	239 (78.10%)	0.099
	Yes	67 (21.89%)	
Number of children	0	218 (71.24%)	0.486
	1	52 (16.99%)	
	2	30 (9.80%)	
	3	6 (1.96%)	

Chi-square test, Pearson.

**Table 3:** Demographic features and BMI of patients.

Vitamin D	Age (years)	Marriage (years)	Years (Childless)	BMI (kg/m^2^)
Low (N = 146)	30 (26–36)	3 (2–5)	2 (1–4)	24.55 (22.71–27.11)
High (N = 160)	30 (28–35)	4 (2–6)	2 (1–4)	25.15 (23.77–27.67)
*p* value	0.365	0.472	0.809	0.071

median (Q1–Q3), BMI: body mass index, Mann Whitney U test.

**Table 4:** Analysis of semen parameters in patients by level of vitamin D.

Vitamin D	Sperm Concentration (million/mL)	Semen Volume (mL)	Total Sperm Count (million/mL)	Total Count Progressive Motile Sperm (million/mL)	AB Motility(%)	C Motility(%)	D Motility(%)	Abstinence Time (day)
Low (N = 146)	28 (7–55)	2.8 (2–4)	70.5 (16–142.75)	28.5 (2.35–76.25)	50 (25–60.5)	6 (5–13)	43 (30–60)	3 (3–4)
High (N = 160)	33 (4.65–65.00)	2.6 (2.0–3.5)	70.0 (12.25–174)	33 (3.22–93)	50.5 (31.5–69.5)	5 (5–10)	38 (25–55)	3 (3–4)
*p* value	0.658	0.377	0.813	0.436	0.022	0.57	0.039	0.489

median (Q1–Q3), Mann–Whitney U test.

**Table 5:** Hormonal and folic acid values of patients by level of vitamin D.

Vitamin D	TSH (mU/L)	FSH (U/L)	LH (U/L)	Prolactin (µg/L)	E2 (ng/L)	Testosterone (ng/mL)	Folic Acid (ng/mL)
Low (N = 146)	1.63 (1.10–2.17)	5.81 (4.15–8.24)	6.12 (4.12–7.59)	14.51 (10.96–17.96)	22.03 (18.03–29.72)	4.15 (3.21–5.45)	6.77 (5.47–8.36)
High (N = 160)	1.53 (1.12–2.1)	5.57 (3.58–8.55)	6.16 (4.95–8.25)	16.11 (11.85–19.68)	23.16 (17.58–30.32)	4.59 (3.36–6.13)	6.84 (5.14–8.66)
*p* value	0.439	0.557	0.277	0.023	0.642	0.104	0.896

median (Q1–Q3), TSH: thyroid stimulation hormone, FSH: follicle-stimulating hormone, LH: luteinizing hormone, E2: estradiol, Mann–Whitney U test.

**Table 6:** Biochemical and blood parameters of patients by level of vitamin D.

Vitamin D	Iron (µg/dL)	Ferritin (µg/L)	Hemoglobin (g/dL)	Hematocrit (g/dL)	Calcium (mg/dL)	Magnesium (mg/dL)	Phosphorus (mg/dL)
Low (N = 146)	87 (78–108.25)	114.5 (76.25–150)	16.3 (15.77–16.7)	47.6 (45.17–48.9)	9.59 (9.24–9.8)	1.96 (1.79–2.16)	3.41 (3.25–3.6)
High (N = 160)	89 (74–116)	98 (74–155)	16.3 (15.6–16.6)	47.6 (46–49)	9.635 (9.5–9.8)	1.99 (1.84–2.17)	3.45 (3.23–3.7)
*p* value	0.753	0.457	0.310	0.597	0.091	0.382	0.797

median (Q1–Q3), Mann–Whitney U test.

**Table 7:** Correlation of level of vitamin D and demographic parameters.

		Age	Marriage	Years (Childless)	BMI
Vitamin D	r_s_	0.068	0.104	0.048	0.107
	P	0.234	0.070	0.403	0.063
	N	306	306	306	306

Spearman’s correlation, r_s_: correlation coefficient, P: significance, N: case number. BMI: body mass index.

**Table 8:** Correlation of vitamin D and semen parameters.

		Sperm Concentration (million/mL)	Semen Volume (mL)	Total Sperm Count (million)	AB Motility (%)	C Motility (%)	D Motility (%)	Progressive Motile Sperm Count (million)	Abstinence Time
Vitamin D	r_s_	0.013	−0.071	−0.036	0.143	−0.134	−0.133	0.035	0.081
	P	0.815	0.218	0.531	0.012	0.019	0.020	0.542	0.156
	N	306	306	306	306	306	306	306	306

Spearman’s correlation, r_s_: correlation coefficient, P: significance, N: case number.

**Table 9:** Correlation of vitamin D, hormones and folic acid.

		TSH (mU/L)	FSH (U/L)	LH (U/L)	Prolactin (µg/L)	E2 (ng/L)	Testosterone (ng/mL)	Folic Acid (ng/mL)
Vitamin D	r_s_	−0.083	0.011	0.101	0.088	0.061	0.135	0.048
	P	0.147	0.847	0.078	0.123	0.285	0.018	0.407
	N	306	306	306	306	306	306	306

Spearman’s correlation, r_s_: correlation coefficient, P: significance, N: case number. TSH: thyroid stimulation hormone, FSH: follicle-stimulating hormone, LH: luteinizing hormone, E2: estradiol.

**Table 10:** Correlation of vitamin D and iron-related and essential mineral parameters.

		Iron (µg/dL)	Ferritin (µg/L)	Hemoglobin (g/dL)	Hematocrit (g/dL)	Calcium (mg/dL)	Magnesium (mg/dL)	Phosphorus (mg/dL)
Vitamin D	r_s_	−0.022	−0.070	−0.027	0.097	0.111	0.065	−0.002
	P	0.707	0.224	0.643	0.092	0.052	0.254	0.974
	N	306	306	306	306	306	306	306

Spearman’s correlation, r_s_: correlation coefficient, P: significance, N: case number.

## Data Availability

All data in this study are included in the manuscript.
